# Clinical Outcomes of the Sushi Technique in Off-Pump Coronary Artery Bypass Grafting for Patients With Aortic Disease: A Propensity Score-Matched Analysis

**DOI:** 10.7759/cureus.100468

**Published:** 2025-12-31

**Authors:** Ryohei Ushioda, Hideki Isa, Dit Yoongtong, Jaroen Cheewinmethasiri, Boonsap Sakboon, Nuttapon Arayawudhikul, Hiroyuki Kamiya

**Affiliations:** 1 Cardiac Surgery, Asahikawa Medical University, Asahikawa, JPN; 2 Surgery, Lampang Hospital, Lampang, THA

**Keywords:** aortic disease, a propensity score-matched analysis, new stroke, off-pump coronary artery bypass grafting, sushi technique

## Abstract

Objectives: Typically, the saphenous vein graft (SVG) is directly anastomosed to the aorta using a side-clamp in off-pump coronary artery bypass grafting (OPCAB). However, in patients with aortic disease, an anastomosis assist device is used, and in some cases, an additional vein graft is anastomosed onto the primary SVG, a technique we refer to as the Sushi technique.

Methods: We retrospectively analyzed 433 OPCAB patients who underwent left internal mammary artery to left anterior descending bypass with aorta-SVG (Ao-SVG) bypass from April 2011 to July 2024. Patients were divided into the Sushi group (n=56) and Ao-SVG group (n=377). A 1:1 propensity score matching was performed (Sushi n=56; Ao-SVG n=56). The Sushi technique was used in patients with aortic disease detected by preoperative CT or intraoperative echocardiography, whereas multiple Ao-SVG anastomoses were performed in those with a healthy aorta.

Results: There were 39 (69.6%) and 195 (51.7%) males, and the mean age was 66.9±8.7 and 66.4±8.4 years in the unmatched cohort, and 39 (69.6%) and 38 (67.9%) males and 66.9±8.7 and 67.3±8.5 years in the matched cohort in the Sushi and Ao-SVG groups, respectively. Stroke rates were comparable (3.6% vs. 1.6%, p=0.28; matched: 3.6% vs. 1.8%, p=1.00). There were no significant differences in major adverse cardiac or cerebrovascular event (MACCE)-free survival (p=0.228) or overall survival (p=0.783).

Conclusion: The new stroke rates did not differ between the groups, suggesting that the Sushi technique could be a viable strategy for patients with aortic disease. Additionally, there was no significant difference between the two groups at five-year free of MACCE or survival rates.

## Introduction

In off-pump coronary artery bypass grafting (OPCAB), the proximal anastomosis of the saphenous vein graft (SVG) has traditionally been performed using a side-clamp on the ascending aorta. However, this technique has been reported to increase the risk of embolic stroke in patients with atherosclerotic plaques or calcification of the ascending aorta [1,2]. In such high-risk cases, an anastomotic assist device, such as the Heartstring (Maquet Holding B.V. & Co. KG, Rastatt, Germany), is sometimes used to avoid clamping the aorta during proximal anastomosis [3].

The “Sushi technique” refers to a composite graft strategy specifically employed when using such proximal anastomotic devices. In cases requiring multi-vessel coronary revascularization, a secondary SVG is anastomosed to a primary SVG, allowing multiple distal targets to be perfused from a single proximal anastomosis site. This approach enables multi-vessel bypass while minimizing aortic manipulation, offering both flexibility and reproducibility in graft configuration.

The Sushi technique is primarily applied in patients with diseased or fragile ascending aortas, in whom minimizing aortic manipulation is essential to reduce the risk of embolic complications. Although this technique is widely used in such high-risk patients, evidence directly comparing its neurologic safety with that of the conventional side-clamping technique remains limited.

The primary endpoint of this study was the incidence of postoperative stroke comparing the Sushi technique with the conventional side-clamping technique. The secondary endpoints included postoperative complications and five-year outcomes such as major adverse cardiac or cerebrovascular events (MACCEs) and overall survival.

## Materials and methods

Study design

This was a retrospective observational study conducted at Lampang Hospital. The study was approved by the Lampang Human Research Ethics Committee of Lampang Hospital (Approval No. 013/67), which waived the requirement for written informed consent owing to the retrospective nature of the study. A total of 2548 patients who underwent isolated OPCAB from April 2011 to July 2024 were identified from our operative database. Among them, we retrospectively analyzed 433 patients who underwent left internal mammary artery to left anterior descending artery grafting combined with SVG to other coronary territories. These patients were divided into two groups according to grafting strategy: the Sushi group (n=56), which employed an anastomotic device, and the aorta-SVG (Ao-SVG) group (n=377), which used a conventional side-clamp technique. The surgical strategy was selected based on the condition of the ascending aorta, assessed by preoperative computed tomography (CT) or intraoperative epiaortic echocardiography. The Sushi technique was applied in patients with suspected aortic disease, while the side-clamp technique was used in those with a healthy aorta. Aortic disease was defined as moderate to severe calcification or atheroma of the ascending aorta on imaging. The Sushi group was propensity score-matched (PSM) with the Ao-SVG group at a 1:1 ratio (Sushi, n=56; Ao-SVG, n=56), and matching was performed based on nine covariates of preoperative clinical characteristics.

**Figure 1:**
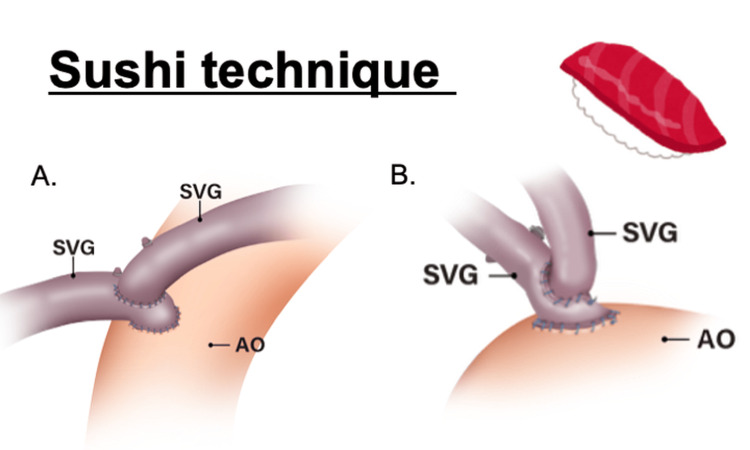
Illustration of the Sushi technique: (A) right and left grafts; (B) V-composite grafts The figure schematically illustrates the concept of the Sushi technique used in off-pump coronary artery bypass grafting. The figure demonstrates the use of a proximal anastomotic assist device to create a single proximal inflow on the ascending aorta (Ao). A primary saphenous vein graft (SVG) is anastomosed at this site, and a secondary SVG is subsequently anastomosed to the primary graft, enabling multi-vessel revascularization while minimizing aortic manipulation and avoiding side-clamping. Credit: Image created by Gosei Aoshima, professional medical illustrator, using Adobe Illustrator (Adobe Inc., San Jose, CA, USA).

The configurations of the Sushi technique are illustrated in Figure 1. All SVGs were harvested using the conventional skeletonized technique and dilated prior to anastomosis. A stroke was defined as the occurrence of a new focal neurological deficit verified by both clinical assessment and CT during hospitalization. Events occurring after discharge were categorized as part of MACCE. Postoperatively, patients were discharged on dual antiplatelet therapy.

Statistical analysis

Group assignments were not randomized, as the operative approach was determined by the surgeon’s subjective choice. To address potential confounding, we calculated the standardized mean difference (SMD) before and after PSM to assess the balance of baseline characteristics between groups. Propensity scores (PS) were derived through logistic regression incorporating nine baseline variables to adjust for potential confounders: age, sex, diabetes mellitus, peripheral arterial disease, chronic obstructive pulmonary disease, cerebrovascular disease, low ejection fraction, elective case, and urgent case. Patients were matched 1:1 using the nearest neighbor method without replacement, applying a caliper width of 0.2 times the standard deviation of the logit of the estimated PS. Parametric continuous variables were expressed as mean ± standard deviation, while non-parametric data were reported as median with range. For comparisons of continuous variables with a normal distribution, the independent Student’s t-test was used in the unmatched cohort and the paired t-test in the matched cohort. For continuous variables with a non-normal distribution, the Mann-Whitney U-test was applied in the unmatched cohort and the Wilcoxon signed-rank test in the matched cohort. Categorical variables were compared using Fisher’s exact test or chi-square test in the unmatched cohort, and McNemar’s test in the matched cohort. Statistical significance was defined as a p-value < 0.05. Univariable and multivariable Cox proportional hazards regression analyses were performed to identify independent risk factors for long-term MACCE, with results expressed as hazard ratio (HR) and 95% confidence interval (CI). Variables with a p-value < 0.05 in the univariable analysis were included in the multivariable model. Prior to multivariable analysis, multicollinearity among independent variables was evaluated. Overall and MACCE-free survival were evaluated using the Kaplan-Meier method. All analyses were conducted with Stata/MP version 18.0 (StataCorp LLC, College Station, TX, US).

## Results

Preoperative data

Table 1 shows preoperative patient characteristics. There were 39 (69.6%) and 195 (51.7%) males, and the mean age was 66.9±8.7 and 66.4±8.4 years in the Sushi and Ao-SVG groups, respectively, in the unmatched cohort. In the matched cohort, both groups included 39 (69.6%) and 38 (67.9%) males, with a mean age of 66.9±8.7 and 67.3±8.5 years, respectively. The median Society of Thoracic Surgeons score was 2.9 (1.7-5.2) in the Sushi group and 2.1 (1.2-3.7) in the Ao-SVG group before matching, while the European System for Cardiac Operative Risk Evaluation II (EuroSCOREⅡ) was 3.8 (1.6-6.2) and 2.4 (1.4-4.2), respectively. After matching, patient backgrounds, including risk scores, were well balanced between the two groups (SMD<10%).

**Table 1:** Patient’s characteristics and preoperative data BMI: body mass index, STS: Society of Thoracic Surgeons, EuroSCORE: European System for Cardiac Operative Risk Evaluation, COPD: chronic obstructive pulmonary disease, CVA: cerebrovascular disease, PAD: peripheral arterial disease, STEMI: ST-elevation myocardial infarction, SMD: standardized mean difference, PS: propensity score, Ao-SVG: aorta-saphenous vein graft

Variable	Entire cohort			PS-matched cohort		
	Sushi group (n=56）	Ao-SVG group (n=377)	SMD	Sushi group (n=56）	Ao-SVG group (n=56)	SMD
Age, mean±SD years	66.9±8.7	66.4±8.4	0.09	66.9±8.7	67.3±8.5	-0.05
Male gender, n (%)	39 (69.6)	195 (51.7)	0.40	39 (69.6)	38 (67.9)	0.04
BMI, mean±SD	23.0±4.0	23.0±3.8	-0.01	23.0±4.0	22.7±3.7	0.01
STS score, median (IQR)	2.9 (1.7-5.2)	2.1 (1.2-3.7)	0.34	2.9 (1.7-5.2)	3.2 (1.8-5.5)	-0.05
EuroSCOREⅡ, median (IQR)	3.8 (1.6-6.2)	2.4 (1.4-4.2)	0.30	3.8 (1.6-6.2)	3.7 (1.8-6.8)	-0.02
Comorbidity, n (%)						
Hypertension	54 (96.4)	372 (98.7)	-0.15	54 (96.4)	54 (96.4)	0.00
Dyslipidemia	54 (96.4)	364 (96.6)	-0.01	54 (96.4)	53 (94.6)	0.09
Diabetes mellitus	32 (57.1)	172 (45.6)	0.18	32 (57.1)	30 (53.6)	0.07
Dialysis	15 (26.8)	84 (22.3)	0.03	15 (26.8)	17 (30.4)	-0.08
COPD	11 (19.6)	31 (8.2)	0.34	11 (19.6)	9 (16.1)	0.09
CVA	6 (10.7)	19 (5.0)	0.22	6 (10.7)	6 (10.7)	0.00
PAD	20 (35.7)	60 (15.9)	0.48	20 (35.7)	19 (33.9)	0.04
STEMI	8 (14.3)	52 (13.8)	0.02	8 (14.3)	8 (14.3)	0.00
Double vessel disease	4 (7.1)	17 (4.5)	0.12	4 (7.1)	4 (7.1)	0.00
Triple vessel disease	52 (92.9)	356 (94.4)	-0.07	52 (92.9)	52 (92.9)	0.00
Ejection fraction <40%	22 (39.3)	115 (30.5)	0.17	22 (39.3)	21 (37.5)	0.04
Urgency, n (%)						
Elective	32 (57.1)	289 (76.7)	-0.44	32 (57.1)	29 (51.8)	0.10
Urgent	24 (42.9)	91 (24.1)	0.42	24 (42.9)	26 (46.4)	-0.07

Intraoperative data

Table 2 shows operative data. There were no significant differences in operative time, number of distal anastomoses, or the use of the sequential technique between the Sushi and Ao-SVG groups in both the unmatched and matched cohorts. However, the rate of complete revascularization was significantly higher in the Ao-SVG group both before and after matching (87.3% vs. 73.2%, p=0.005; after matching: 89.2% vs. 73.2%, p=0.023).

**Table 2:** Intraoperative short-term outcomes * Mann-Whitney U test, ** Wilcoxon rank-sum test, ^#^ Chi-square test, ^##^ Fisher’s exact test, ^###^ McNemar’s test Z: test statistic for nonparametric test, χ^2^: chi-square value, CPB: cardiopulmonary bypass, Ao-SVG: aorta-saphenous vein graft, PS: propensity score, IQR: interquartile range

Variable	Entire cohort				PS-matched cohort			
	Sushi group (n=56）	Ao-SVG group (n=377)	P-value	Test statistic	Sushi group (n=56）	Ao-SVG group (n=56)	P-value	Test statistic
Operating time, median (IQR) min	212.5 (192.5-246)	220 (195-250)	0.46*	Z=0.73	212.5 (192.5-246)	225 (195-252.5)	0.89**	Z=-0.14
Total graft number, median (min-max)	3 (3-4)	3 (3-5)	0.82*	Z=0.23	3 (3-4)	3 (3-4)	0.63**	Z=-0.48
Number of distal anastomoses, median (min-max)	3 (3-5)	3 (3-5)	0.54*	Z=-0.61	3 (3-5)	3 (3-5)	0.53**	Z=-0.63
Endarterectomy, n (%)	1 (1.8)	3 (0.8)	0.44^##^	Fisher’s exact test	1 (1.8)	1 (1.8)	1.00^###^	McNemar’s test
Complete revascularization, n (%)	41 (73.2)	329 (87.3)	0.005^#^	χ^2^=7.75	41 (73.2)	50 (89.2)	0.023^###^	χ^2^=5.15
Conversion to CPB, n (%)	2 (3.6)	10 (2.7)	0.66^##^	Fisher’s exact test	2 (3.6)	3 (5.4)	1.00^###^	McNemar’s test
The sequential technique of vein graft, n (%)	22 (39.3)	134 (35.5)	0.69^#^	χ^2^=0.16	22 (39.3)	18 (32.1)	0.55^###^	χ^2^=0.36

Postoperative data

Table 3 shows postoperative short-term outcomes. In the unmatched cohort, the incidence of new stroke was 3.6% in the Sushi group and 1.6% in the Ao-SVG group (p=0.28). After PS matching, the stroke rate remained similar between the two groups (3.6% vs. 1.8%, p=1.00), with no significant difference observed. Other early outcomes, including intensive care unit and hospital stay, early extubation, and 30-day mortality, were comparable between the groups both before and after matching. There were no significant differences in other postoperative major complications.

**Table 3:** Postoperative short-term outcomes * Mann-Whitney U test, ** Wilcoxon rank-sum test, ^#^ Chi-square test, ^##^ Fisher’s exact test, ^###^ McNemar’s test Z: test statistic for nonparametric test, χ^2^: chi-square value, ICU: intensive care unit, PS: propensity score, Ao-SVG: aorta-saphenous vein graft, IQR: interquartile range

Variable	Entire cohort				PS-matched cohort			
	Sushi group (n=56）	Ao-SVG group (n=377)	p-value	Test statistic	Sushi group (n=56）	Ao-SVG group (n=56)	p-value	Test statistic
Median ICU stay (IQR), days	2 (1-3)	2 (1-2)	0.09*	Z=-1.71	2 (1-3)	2 (1-4.5)	0.45**	Z=-0.76
Median hospital stay (IQR), days	5.5 (5-7)	5 (5-6)	0.55*	Z=-0.59	5.5 (5-7)	6 (5-8)	0.23**	Z=-1.21
Early extubation (≦24h), n (%)	49 (87.5)	332 (88.1)	1.00^#^	χ^2^=0.01	49 (87.5)	47 (83.9)	0.79^###^	χ^2^=0.07
30-day mortality, n (%)	2 (3.6)	10 (2.7)	0.66^##^	Fisher’s exact test	2 (3.6)	4 (7.1)	0.69^###^	McNemar’s test
Early term postoperative complications, n (%)								
New stroke	2 (3.6)	6 (1.6)	0.28^##^	Fisher’s exact test	2 (3.6)	1 (1.8)	1.00^###^	McNemar’s test
New dialysis	0 (0)	3 (0.8)	1.00^##^	Fisher’s exact test	0 (0)	3 (5.4)	0.25^###^	McNemar’s test
New onset atrial fibrillation/flutter	18 (32.1)	114 (30.2)	0.77^#^	χ^2^=0.08	18 (32.1)	22 (39.3)	0.57^###^	χ^2^=0.32
Infection of the wound	1 (1.8)	4 (1.1)	0.50^##^	Fisher’s exact test	1 (1.8)	0 (0)	1.00^###^	McNemar’s test
Reoperation for bleeding	2 (0)	3 (3.6)	0.13^##^	Fisher’s exact test	2 (3.6)	0 (0)	0.50^###^	McNemar’s test

**Figure 2:**
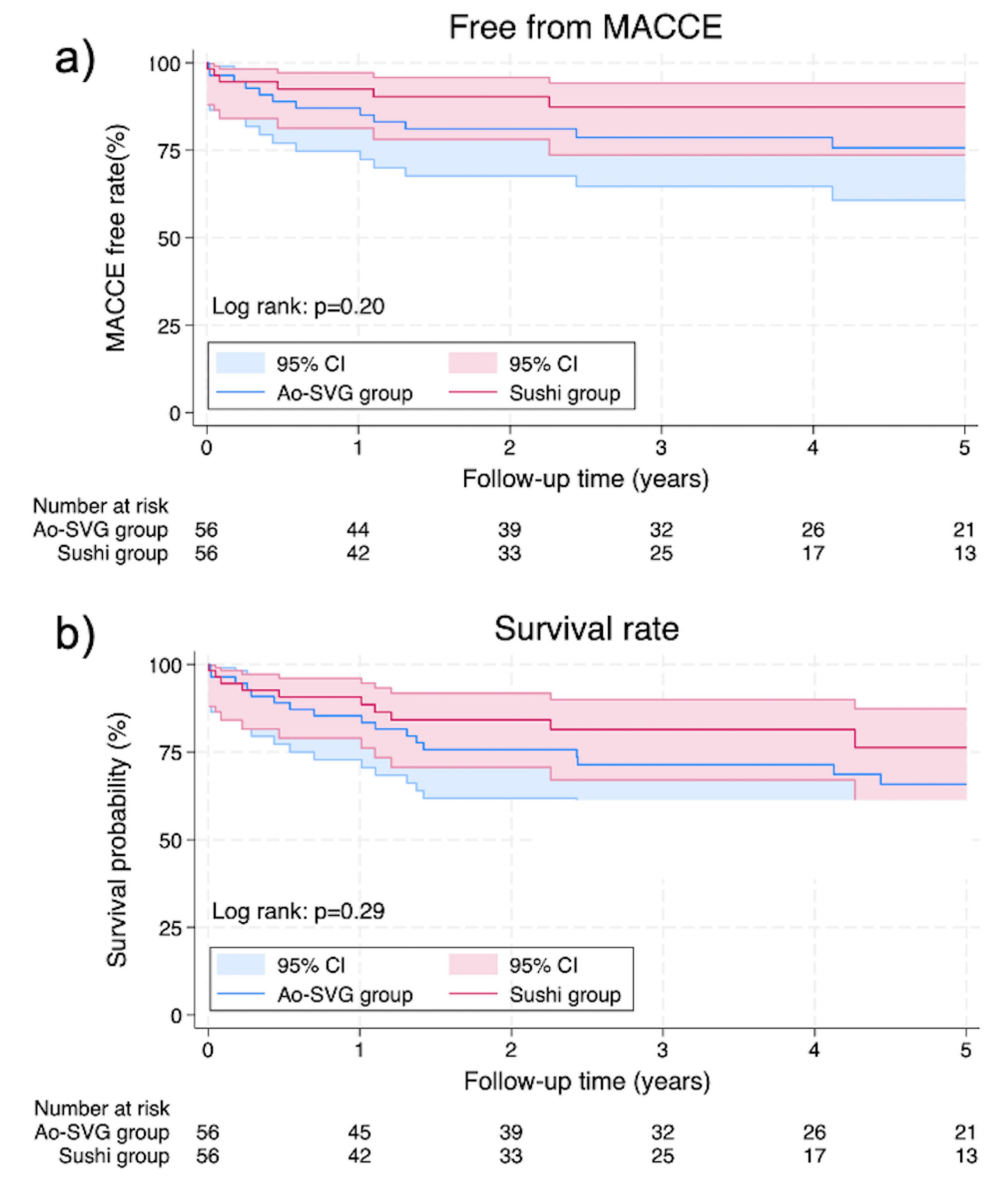
Kaplan-Meier curves showing (A) freedom from MACCE (p=0.20) and (B) overall survival (p=0.29) at five years after surgery MACCE: major adverse cardiac and cerebrovascular events, Ao-SVG: aorta-saphenous vein graft, CI: confidence interval

The Kaplan-Meier curve of the postoperative free-from MACCE rate and survival rate is shown in Figure 2. There is no significant difference in MACCE (p=0.20) and survival rate (p=0.29) between the two groups.

Table 4 shows the results of univariable and multivariable Cox regression analyses for factors associated with MACCE. In the multivariable analysis, older age (HR: 1.03, 95% CI: 1.00-1.06, p=0.041) and dialysis dependence (HR: 2.22, 95% CI: 1.36-3.62, p=0.001) were identified as independent risk factors for MACCE. Other preoperative variables, including diabetes mellitus, peripheral arterial disease, and low ejection fraction, were not significantly associated with MACCE in the multivariable model. The type of graft (Sushi vs. Ao-SVG) was not associated with MACCE.

**Table 4:** Univariable and multivariable analyses of factors associated with MACCE MACCE: major adverse cardiac or cerebrovascular events, HR: hazard ratio, CI: confidence interval, COPD: chronic obstructive pulmonary disease, CVA: cerebrovascular accident, PAD: peripheral arterial disease, STEMI: ST-elevation myocardial infarction, EF: ejection fraction, NA: not applicable

Variable	Univariable analysis			Multivariable analysis		
	HR	95% CI	P-value	HR	95% CI	P-value
Preoperative factor						
Age	1.03	1.00-1.06	0.028	1.03	1.00-1.06	0.041
Male gender	0.98	0.62-1.53	0.93	-	-	-
Hypertension	1.02	0.14-7.33	0.99	-	-	-
Dyslipidemia	0.97	0.24-3.96	0.97	-	-	-
Diabetes mellitus	1.04	0.66-1.64	0.86	-	-	-
Dialysis	2.35	1.45-3.80	<0.001	2.22	1.36-3.62	0.001
COPD	1.38	0.71-2.69	0.34	-	-	-
CVA	1.50	0.60-3.71	0.39	-	-	-
PAD	1.63	0.95-2.80	0.08	1.31	0.75-2.30	0.34
STEMI	0.76	0.38-1.53	0.45	-	-	-
Double vessel disease	1.32	0.53-3.28	0.55	-	-	-
Triple vessel disease	0.59	0.27-1.28	0.18	-	-	-
Low EF (<40%)	1.31	0.82-2.09	0.27	-	-	-
Elective	0.58	0.36-0.95	0.03	NA	NA	NA
Urgent	1.78	1.10-2.90	0.02	1.46	0.88-2.42	0.14
Intraoperative factors						
Operation time	1.00	0.99-1.00	0.70	-	-	-
Sushi technique	0.89	0.41-1.93	0.76	-	-	-
Complete revascularisation	2.38	0.73-7.68	0.15	-	-	-

## Discussion

Sushi technique and neurologic safety in aortic disease

In the present study, the Sushi technique was applied exclusively to patients with aortic disease, whereas the conventional side-clamp method was used only in those with a healthy ascending aorta. Despite this clear difference in baseline risk, the incidence of new-onset postoperative stroke was similarly low in both groups after PS matching (3.6% vs. 1.8%, p=1.00). This finding suggests that the Sushi technique, which employs the Heartstring proximal anastomotic device, provides neurologic safety comparable to the standard approach, even in anatomically high-risk patients.

Aortic manipulation, particularly the use of conventional side-clamping during proximal graft anastomosis, is a well-recognized risk factor for perioperative stroke in coronary artery bypass surgery [1,2]. To eliminate this risk, anaortic OPCAB techniques have been developed to completely avoid aortic contact [4]. However, due to their technical demands, such methods have not been widely adopted in routine surgical practice. The Sushi technique, while not entirely anaortic, substantially reduces aortic handling by allowing for a single proximal anastomosis without side-clamping. This strategy offers a practical compromise between procedural simplicity and neurologic protection.

Multiple studies over the past decade have supported the benefits of minimizing aortic manipulation. The previous studies have demonstrated that Heartstring-assisted OPCAB achieved stroke rates comparable to those of anaortic OPCAB, and significantly lower than those observed in on-pump coronary artery bypass [5,6]. Our results are consistent with these findings and suggest that the Sushi technique, when applied with proper indications and technique, represents a safe and effective alternative for high-risk patients in whom aortic side-clamping is undesirable. Collectively, these findings highlight the clinical utility of the Sushi technique as a viable strategy for maintaining neurologic safety in patients with diseased aortas, while also preserving surgical flexibility and reproducibility.

Composite graft configuration in the sushi technique

The Sushi technique is a collective term encompassing various composite graft configurations, including V-composite grafts and right-and-left grafts. These configurations enable multi-vessel revascularization through a single proximal inflow, minimizing the number of aortic anastomoses and reducing aortic manipulation - an advantage in patients with aortic disease.

Several studies have demonstrated the clinical utility and structural validity of such composite graft strategies. Nishigawa et al. [7] reported a V-composite graft using a free right internal mammary artery (RIMA) anastomosed to a short aorto-coronary vein graft within 1 cm of the aorta, which achieved 97.6% early patency and 93.6% one-year patency. Similarly, right-and left-two-limb composite grafts have shown favorable mid-term patency and clinical outcomes [8]. Yoshizumi et al. [9] also demonstrated that a modified proximal anastomosis technique using a free RIMA and a small interposed graft segment provided outcomes comparable to in situ RIMA, while allowing for increased target reach and flexibility. Collectively, these findings support the use of single-inflow composite grafting - as exemplified by the Sushi technique - as a practical and versatile strategy for complex coronary revascularization, particularly in patients requiring reduced aortic manipulation.

Limitation

The present study had several limitations. First, the present investigation is a retrospective, non-randomized analysis using data from a single medical center. Second, we performed PSM based on the different characteristics of the study patients before the operation. Nevertheless, there were several unmeasured confounders. Third, preoperative neurological evaluations, including brain magnetic resonance imaging (MRI) or CT, were not consistently performed in all patients. Fourth, asymptomatic strokes might have been missed because routine postoperative brain imaging was not conducted. Finally, follow-up data on SVG patency were not available, and it remains unclear whether the Sushi technique can demonstrate SVG patency rates comparable to those of independent aorta-coronary (A-C) bypass.

## Conclusions

The Sushi technique appears to be a safe and feasible surgical strategy for patients with diseased or fragile ascending aortas, providing multi-vessel revascularization with minimal aortic manipulation. This approach avoids side-clamping and may reduce neurological risk without compromising graft efficacy. Further prospective multicenter studies are warranted to validate long-term patency, stroke prevention, and broader clinical utility.
